# The application of the tracer method with peer observation and formative feedback for professional development in clinical practice: a scoping review

**DOI:** 10.1007/s40037-021-00693-6

**Published:** 2021-11-11

**Authors:** Rudi A. Steenbruggen, Marjo J. M. Maas, Thomas J. Hoogeboom, Paul L. P. Brand, Philip J. van der Wees

**Affiliations:** 1 School of Health, Physiotherapy, Saxion University of Applied Sciences, Enschede, The Netherlands; 2 Radboud Institute for Health Sciences, IQ Healthcare, Radboud University Medical Center, Nijmegen, The Netherlands; 3 Institute of Allied Health Studies, HAN University of Applied Sciences, Nijmegen, The Netherlands; 4 Isala Hospital, Zwolle, The Netherlands; 5 University of Groningen and University Medical Centre, Groningen, The Netherlands

**Keywords:** Learning environment, Learning style, Evaluation

## Abstract

**Introduction:**

The tracer method, commonly used for quality assessment, can also be used as a tool for peer observation and formative feedback on professional development. This scoping review describes how, by whom, and with what effect the tracer method is applied as a formative professional development instrument between healthcare professionals of equal status and aims to identify the types of scientific evidence for this use of the tracer method.

**Methods:**

The authors searched four electronic databases for eligible articles, which were screened and assessed for eligibility by two independent researchers. From eligible studies, data were extracted to summarize, collate, and make a narrative account of the findings.

**Results:**

The electronic search yielded 1757 unique studies, eight of which were included as valid and relevant to our aim: five qualitative, two mixed methods, and one quantitative study. Seven studies took place in hospitals and one in general practice. The tracer method was used mainly as a form of peer observation and formative feedback. Most studies evaluated the tracer method’s feasibility and its impact on professional development. All but one study reported positive effects: participants described the tracer method generally as being valuable and worth continuing.

**Discussion:**

Although the body of evidence is small and largely limited to the hospital setting, using the tracer method for peer observation and formative feedback between healthcare professionals of equal status appears sufficiently useful to merit further rigorous evaluation and implementation in continuous professional development in healthcare.

**Supplementary Information:**

The online version of this article (10.1007/s40037-021-00693-6) contains supplementary material, which is available to authorized users.

## Introduction

The tracer method was introduced in 1973 as a tool to assess the quality of care provided in a healthcare system [1]. Over the years, the tracer method has gained increasing worldwide popularity as an evaluation and assessment method to document a patient’s experience, a healthcare process or product, resulting in both summative and formative feedback for accreditation of health services [2–5]. Carrying out this form of auditing is associated with improved patient experiences and observed safety on hospital wards, with no adverse outcomes on safety culture and team climate [6]. It has been proposed that the tracer method could also be a useful strategy to support continuous professional development of healthcare professionals in clinical practice [7, 8]. During a tracer, a professional quality assessor or peer assessor (colleague of equal status) observes a healthcare professional during daily practice and provides feedback on a number of pre-established indicators [9]. Using the method in this way can be viewed as a form of peer observation and formative feedback. A systematic review provides theoretical support for the use of audit and feedback in professional practice and healthcare, by showing that feedback is more likely to be effective if the feedback provider is a supervisor or a colleague, and when it is provided more than once, delivered in verbal and written formats, and when it includes explicit targets and an action plan [10]. However, a thorough overview of the evidence of the effects of the tracer method with peer observation and merely formative feedback, carried out by a colleague of equal status, primarily focused on continuous professional development purposes in healthcare, is lacking. Recent reviews of the tracer method in healthcare quality management found little evidence of using the tracer method outside the scope of accreditation of health service organizations [8, 9]. Thus, we conducted this scoping review based on the following research question: How, by whom, and with what effect is the tracer method with peer observation and formative feedback applied as an instrument for professional development of healthcare professionals?

## Method

We conducted a scoping review according to the guidance of Arksey and O’Malley, Levac, Grant, and the JBI Guide for scoping reviews [11–14]. The first and second author conducted all the steps of the review, the others critically appraised the research process and provided feedback. Following the methodological framework of conducting a scoping review, every next step was only taken after achieving consensus by the whole research team. We report our findings in accordance with the PRISMA Extension for Scoping Reviews (PRISMA-ScR) [15].

During the first stage, the key aspects of the study objective were translated into the research question as described. The research question guided the search strategy and the following steps of the review.

In January 2020, with an update in January 2021, we searched four databases (CINAHL, Cochrane, Embase and MEDLINE) from the inception, in collaboration with a university librarian, using the search terms along with their most important synonyms and alternative definitions, as generated from the research question (See Appendix 1 of the Electronic Supplementary Material for the full search strategies).

We searched Google Scholar for grey literature in February 2020 limiting our inclusion to the first 100 hits, because the relevance of retrieved studies declined sharply afterwards. In addition, we asked JCI, Qmentum (the two global market leaders on identifying, measuring and sharing best practices in quality and patient safety in hospitals) and an expert in the field (P. Lalleman, the Netherlands) what they considered to be the most important literature on the topic.

Articles were screened on relevance by title and abstract by two independent reviewers (RS and MM), using prespecified inclusion and exclusion criteria. As peer observation programs globally do not always refer to the tracer method, we transformed our comprehensive set of relevant terms into an extensive set of criteria. Only studies with full text available were included if (a) healthcare professionals, peers or colleagues were the subject of the study; (b) when shadowing, tracing, direct observation, feedback peer review and/or peer evaluation was used, and (c) where the purpose was development of competencies, performance and/or quality improvement. Studies with an English abstract and the main text in German, French or Spanish were also included using translation software. Studies were excluded if they took place in an educational setting, if simulation, masked/secret observation and/or video observation was used, where there was a dependent or hierarchical relationship between observer/feedback provider and the observed healthcare professional/feedback recipient, and where the purpose was an audit, certification, assessment and/or examination.

These criteria were chosen to ensure inclusion of studies with a formative purpose (i.e., aiming at continuous professional development or performance improvement) by healthcare professionals. Studies on the use of the tracer method for accreditation purposes or for summative assessment by a supervisor in a hierarchical relation were excluded. Differences in opinion of the two reviewers were resolved by discussion, and when needed consensus was obtained by consulting a third research team member. Then, the full text of the selected articles was screened against the inclusion and exclusion criteria independently by the first and second author (RS and MM), and the reasons for excluding articles were recorded. Covidence software was used to support the study selection process [16].

From the selected articles, the following predefined data were extracted by two reviewers (RS and MM): author(s), year of publication, study location, population, the objective of the study, type of intervention, methodology, results, impact on professional development, perceived barriers and facilitators, and conclusion(s). We did not appraise the quality of the evidence of selected articles, because a scoping review provides a preliminary assessment of the potential size and scope of available research and it aims to identify the nature and extent of research evidence [13, 14].

The extracted data were analyzed based on the research questions, leading to collating, summarizing, and reporting of results. Data from qualitative or mixed methods studies were subjected to thematic analysis, following guidelines from the literature [17, 18], by line-by-line coding by two reviewers (RS and MM) to identify themes. These were included in the results when consensus was reached between reviewers. If needed, a third research team member was consulted. Types of evidence were classified according to Kirkpatrick’s model, a useful method for evaluating training outcomes, consisting of four successive levels of learning effects: reactions, learning, behavior, and results [19].

We invited all first authors of the included studies to join this panel, in order to discuss and validate our findings and to identify any missing information on the topic. After accepting the invitation, participants in the panel were sent the draft version of the review in preparation, together with a zoom link for the set date and time of the panel meeting. Three authors (PvdW, MM, RS) prepared the panel meeting by drawing up an agenda and a topic list. The meeting was chaired by PvdW and introduced on content by RS. The audio recording of the meeting was transcribed and thematically analyzed by the two first authors (RS and MM). Conclusions thus drawn were sent to participants for a member-check. Convening and consulting the expert panel was exempt from medical ethical review under Dutch law. All panel members participated voluntarily.

## Results

**Fig. 1:**
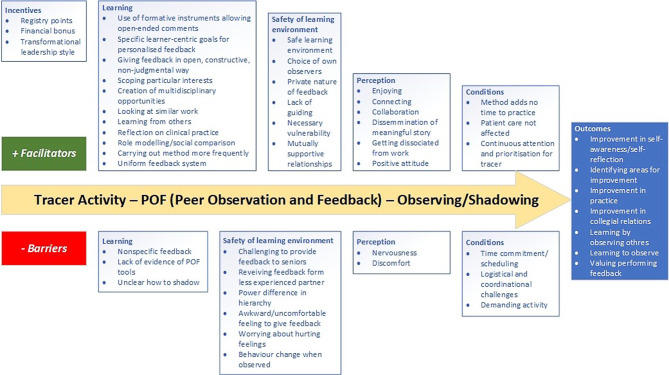
Summary of thematic analysis of studies consisting entirely or partially of a qualitative design regarding carrying out tracer activity

We identified 2300 potential studies of which we included eight ([20–27]; see the Fig. 1 and Table 1 of the Electronic Supplementary Material). All included studies used the tracer method in the form of peer observation and feedback. Three studies used a standardized feedback instrument [20, 21, 24]. Seven of the eight included studies took place in a hospital [20, 22–27], and one in general practice [21]. Overall, the study population of included studies comprised 228 pediatricians (mainly from one study in which 198 pediatricians participated), five other medical specialists, 16 nursing managers, three specialist nurses and two general practitioners. In these studies, the tracer method was used to assess its use in clinical practice (feasibility) and to assess its impact on professional development (effectiveness). The four feasibility studies described healthcare professionals’ experiences with the tracer method as a form of peer observation and feedback [22, 23, 26, 27].

Five studies applied qualitative research methods (interviews or surveys with open-ended questions) [20, 21, 23, 25, 26], two used mixed methods (questionnaires with open and closed questions and the use of the I‑PASS (Illness—Patient—Action—Situation—Synthesis) mnemonic) [22, 24], and one study used quantitative data (survey with questions scored on a Likert scale) [27].

The effectiveness studies aimed to evaluate the change in professional attitude or behavior (*n* = 3) [21, 23, 26] or to investigate whether the instrument used impacted learning and development (*n* = 2) [21, 26]. Secondary study aims included investigating the efficacy of the method, characterizing the practice of peer observation and feedback, and identifying preferences for the use of the method.

All the studies but one reported positive perceived effects: participants described the tracer method as being valuable (*n* = 6) [20, 22–24, 26, 27] and worthy of being repeated (*n* = 2) [22, 24], as an innovative, interesting and effective training supporting professionals’ ongoing learning (*n* = 2) [21, 22], as a tool to strengthen the work culture/collectivity (*n* = 2) [22, 26], as a promoter of growth through collaboration (*n* = 2) [23, 24] and as an instrument to stimulate an investigative attitude among professionals (*n* = 2) [23, 26]. One study concluded that the application of the tracer method did not lead to strong learning effects [25].

One study, covering all four levels of Kirkpatrick’s model, concluded that direct peer observations with feedback strengthened the workplace culture, promoted growth through collaboration, and allowed acceptance and success of future projects involving peer observations and feedback [24]. Two studies covered three levels from reactions to behavior [21, 26], most studies examined only the first two levels of reactions and learning [20, 22, 23, 25], and one study covered only the level of reactions [27].

### Thematic analysis of qualitative data from qualitative studies

Regarding feasibility, we identified five analytical themes: learning, incentives, safety of learning environment, perceptions, and conditions. All these themes can be regarded as either a facilitator or a barrier to the feasibility of the tracer method, finally having impact on its effectiveness on professional development (Fig. 1).

Most of the comments in the qualitative studies were about learning. Regarding the method, the use of questionnaires allowing for written comments, carrying out the tracer method more frequently, and a uniform feedback system were seen as important facilitators for learning [20, 25]. Other perceived advantages of the tracer method as a learning tool included its flexible design depending on personal interests, its ability to create multidisciplinary learning opportunities, and its design, providing space for specific learner-centric goals for personalized feedback [20, 21, 25, 27]. On a more personal level, the art of giving feedback in an open, constructive, non-judgmental way was seen as important [20, 26]. Applying the method in daily practice, where it is possible to look at similar work, to reflect on clinical practice, to learn directly from a colleague and to make a social comparison, were noted as important facilitators for learning [25, 27]. Perceived barriers in using the tracer method for learning were the provision of nonspecific feedback, uncertainty about the implementation of the method, and the absence of evidence on its usefulness [23, 27].

Incentives, such as a financial bonus or the allocation of continuing education credits for a professional quality register, were viewed as facilitators to participate in a program using the tracer method [21]. Application of a transformational leadership style, in which leaders encourage, inspire, and motivate employees to innovate and create change that will help grow and shape their future success, was also seen as an incentive [25].

A safe learning environment was considered to be the most important facilitator for successful application of the tracer method. Participants described a safe learning environment as an environment in which the method could be applied with a sense of freedom, without external guidance, with internal observers and feedback offered privately, in a mutually supportive relationship, acknowledging the vulnerability of the traced professional [20, 24, 26, 27]. Perceived barriers included the awkwardness of providing feedback to a colleague, fear of hurting other people’s feelings, perceived hierarchal differences between feedback provider and recipient, and the potential of people changing their behavior when being observed [20, 22, 27].

A facilitator’s positive experience with the tracer method impacted their perception of it and encouraged a more frequent use of the method. Not only a positive attitude towards the method itself and a desire to use it, but also connecting and collaborating with colleagues, getting away from daily work and disseminating a meaningful story were considered important elements in this respect [23, 24, 26]. We noted nervousness and discomfort to participate in a tracer cycle as barriers for continuing the method [22, 26, 27].

The most important condition for successfully applying the tracer method was time management, which could be both a facilitator and a barrier. Conditions were perceived as facilitators when the method did not take more time in practice and did not affect patient care [22]. By contrast, when the method did not fit into regular patient care scheduling and became a demanding activity with logistical and organizational challenges, this was felt to be a barrier for applying it [20, 22–24, 26, 27]. Another condition mentioned as a facilitator was a climate of ongoing attention and prioritization for tracer activity [25].

Overall, seven positive outcomes of applying the tracer method were noticed in the qualitative studies. Participants reported not only identifying areas for their own professional improvement but commented that this also contributed to improved self-awareness and self-reflection, and to supporting collegial relations [20–24, 26, 27]. Participants also mentioned learning to observe, learning by observing others, and valuing performing feedback as beneficial outcomes of applying the tracer method [23, 26].

### Expert consultation panel

The expert consultation panel consisted of seven participants: four authors of included studies and three authors of this scoping review (RS, MM and PvdW). Overall, participants agreed with the design and the results of the review and believed, linked to their own experiences, that no essential topics were missed. The authors provided feedback to adjust some details of the review, including the representation of the thematic analysis in Fig. 1. Also, the panelists expressed unfamiliarity with the term “tracer method” and suggested to specify this by adding the characteristic “peer observation and feedback”, because they believed this to be of key importance in the effective use of the tracer method for continuous professional development.

## Discussion

This scoping review shows that the application of the tracer method with peer observation and formative feedback for continuous professional development has been studied mainly in hospital settings to assess its feasibility and impact. In all included studies—five qualitative studies, two mixed methods studies, and one quantitative study—the researchers used the peer observation and formative feedback, by medical specialists and general practitioners, and by nurses and nursing managers. The application of the tracer method addressed all four levels of Kirkpatrick’s model (reactions, learning, behavior, results) in only one study. Participants valued applying the tracer method and found it useful for their professional development.

We propose further research should focus on the design and conduct of more extensive, and rigorous studies on the evaluations of the tracer method in continuous professional development in healthcare, especially if the observed facilitators and barriers are sufficiently considered. A good starting point would be to generate more complex evaluation designs resulting in quantitative and qualitative data on the method to gather more robust evidence of its effects. It is conceivable to undertake this research not only in clinical practice but also, for example, in education of healthcare professionals so that already at this stage the basic principles of continuous learning are taught, for which the tracer method with peer observation and formative feedback can be an important basis. Therefore, we argue for tailoring the design and implementation of the instrument to the specific context of healthcare professionals or students. Because direct observation and formative feedback are familiar to most healthcare professionals and students, and the term ‘tracer method’ has a growing reputation through the use of globally applied quality systems such as JCI and Qmentum, existing knowledge and experience in this field could be applied to use the tracer method as a quality improvement instrument for professional performance as well.

In comparison to the literature, this study demonstrates that only a few studies have examined the tracer method as a tool for direct peer observation and formative feedback, applied in a non-dependent and non-hierarchical relationship and for professional development purposes. It has been shown that direct observation of health professional trainees is valid and representative in assessing a broad spectrum of skills and competencies [28]. However, the literature on such direct observation is potentially influenced by the fact that it is unclear whether the direct observation is intended as assessment (summative assessment) or as a source for formative feedback. A growing body of research suggests that this distinction is crucially important [29]. Particularly also the question of whether the learner (observed) perceives the direct observation and feedback as an exam or as an opportunity to learn and grow. Therefore, we deliberately limited ourselves to studies that described that they were aimed at promoting growth and development (formative) and excluded studies that made summative judgments.

In most studies, the tracer method has been used in the context of quality assessment and as an accreditation tool for healthcare organizations [20, 21, 23–27]. Other forms of peer observation and feedback, for instance through indirect observation via video, have been used to improve the quality of healthcare, for example in improving hand hygiene and medical administration [30–34]. Our results agree with these findings, and with those of studies on the feasibility of peer observation and feedback in clinical practice [35, 36]. Our observation that participants consider the application of the method to be valuable has also been confirmed in two studies [37, 38].

Although Cheung et al. did not mention the tracer method explicitly in their study, they identified key barriers and enablers to direct observation and feedback in clinical practice and proposed that discordant intentions between observers and observed, together with social expectations that the observed should be responsible for ensuring that observations occur, may lie at the root of why direct observation and feedback tend to occur so infrequently in practice [39]. Veloski et al. stated that the effects of formal (summative or formative) assessment and feedback on physician performance are positively influenced by the reliability of the source and duration of the feedback [40]. These barriers and facilitators correspond to our findings. Both the evidence and the experience in the field of graduate and postgraduate medical education highlight the need to distinguish feedback being used to guide learners towards growth and development (formative feedback), from assessment that is being used for summative judgement [29]. The results of our thematic analysis suggest that the same applies to using the tracer method as a tool to promote development of professional competencies.

Our study has several limitations. Although we applied a deliberately sensitive search strategy and consulted experts in the field, a scoping review may always miss relevant studies. Where scoping reviews tend to be used to map more extensive bodies of literature to find gaps in existing knowledge, we found only eight studies that met our inclusion criteria. Thus, our conclusions regarding the scope of our review should be interpreted with caution. Following the recommendations of the methodological framework for scoping reviews, we refrained from methodological appraisal of the included studies, because a scoping review provides a preliminary assessment of the potential size and scope of available research and it aims to identify the nature and extent of research evidence, so that the validity of the retrieved evidence was not formally assessed [13, 14].

## Conclusion

Application of the tracer method with peer observation and feedback holds promise as a tool to promote professional development of healthcare professionals because participants value the method to stimulate their learning. Although the evidence is scarce and robust quantitative data are lacking, particularly on the effect of the method on healthcare professionals behavior, the use of the tracer method as a professional development tool by healthcare professionals of equal status tentatively indicates a potential usefulness of the tracer method as a quality improvement instrument.

Because the body of evidence is small and largely limited to the hospital setting, the scope for further research in the early stages of this field should be on the design and conduct of further, more extensive, and rigorous studies on the evaluations of the tracer method in continuous professional development in healthcare, especially if the observed facilitators and barriers are sufficiently considered.

## Supplementary Information

Search strategies for each database on December 30th, 2019

Fig. 1 Flow Chart of the selection process

Table 1 Characteristics of the included studies
