# Supplementary material for: The application of the tracer method with peer observation and formative feedback for professional development in clinical practice: a scoping review
Source: Perspect Med Educ. 2021 Nov 11;11(1):15–21. doi: 10.1007/s40037-021-00693-6 (PMC8733089; doi:10.1007/s40037-021-00693-6)
Supplement: Supplementary file 1 — Search strategies for each database on December 30th, 2019 [file 40037_2021_693_MOESM1_ESM.docx]

**Electronic Supplemental Resource; Search strategies for each database on December 30th, 2019.**

**PubMed**

(peer observation[tiab] OR peer clinical observation[tiab] OR direct observation[tiab] OR shadowing[tiab] OR peer feedback[tiab] OR peer assessment[tiab] OR (("Formative Feedback"[Mesh] OR Peer group[Mesh] OR Peer review[Mesh]) AND Observation[Mesh]))

AND

(profession[tiab] OR pro[tiab] OR expert[tiab] OR professionalism[tiab] OR professional behavio*[tiab] OR health personnel[Mesh])

AND

(competenc*[tiab] OR cognizance[tiab] OR cognisance[tiab] OR knowledge[tiab] OR skill*[tiab] OR attitude*[tiab] OR behavio*[tiab] OR "Health Knowledge, Attitudes, Practice"[Mesh] OR behavior[Mesh] OR professional competence[Mesh])

AND

(perception[tiab] OR perceptions[tiab] OR learning[tiab] OR behavioral change*[tiab] OR behavioural change*[tiab] OR quality performance[tiab] OR organizational performance[tiab] OR organisational performance[tiab]OR patient outcome*[tiab] OR professional development[tiab] OR competentional development[tiab] OR competence development[tiab] OR awareness[tiab] OR "Quality of Health Care"[Mesh] OR "Learning"[Mesh] OR "Awareness"[Mesh])

**Embase**

(peer observation.ti,ab,kw. OR peer clinical observation.ti,ab,kw. OR direct observation.ti,ab,kw. OR shadowing.ti,ab,kw. OR peer feedback.ti,ab,kw. OR peer assessment.ti,ab,kw. OR ((constructive feedback/ OR exp Peer group OR "peer review"/) AND observation/))

AND

(profession.ti,ab,kw. OR pro.ti,ab,kw. OR expert.ti,ab,kw. OR professionalism.ti,ab,kw. OR professional behavio*.ti,ab,kw. OR exp health care personnel/)

AND

(competenc*.ti,ab,kw. OR cognizance.ti,ab,kw. OR cognisance.ti,ab,kw. OR knowledge.ti,ab,kw. OR skill*.ti,ab,kw. OR attitude*.ti,ab,kw. OR behavio*.ti,ab,kw. OR exp health personnel attitude/ OR professional knowledge/ OR behavior/ or behavior change/ OR achievement/ or goal attainment/ or job performance/ or performance/ OR competence/ or clinical competence/ or nursing competence/ or professional competence/)

AND

(perception.ti,ab,kw. OR perceptions.ti,ab,kw. OR learning.ti,ab,kw. OR behavioral change*.ti,ab,kw. OR behavioural change*.ti,ab,kw. OR quality performance.ti,ab,kw. OR organizational performance.ti,ab,kw. OR organisational performance.ti,ab,kw.OR patient outcome*.ti,ab,kw. OR professional development.ti,ab,kw. OR competentional development.ti,ab,kw. OR competence development.ti,ab,kw. OR awareness.ti,ab,kw. OR health care quality/ or benchmarking/ or clinical effectiveness/ or incident report/ or exp medical error/ or nursing outcome/ or exp professional standard/ or "quality of nursing care"/ OR learning/ or collaborative learning/ or constructive feedback/ or lifelong learning/ or self-directed learning/ OR awareness/)

**CINAHL**

(TI (peer observation OR peer clinical observation OR direct observation OR shadowing OR peer feedback OR peer assessment) OR AB (peer observation OR peer clinical observation OR direct observation OR shadowing OR peer feedback OR peer assessment) OR (((MH "Feedback") OR (MH "Peer Group") OR (MH "Peer Review+")) AND (MH "Observational Methods+")))

AND

(TI (profession OR pro OR expert OR professionalism OR professional behavio*) OR AB(profession OR pro OR expert OR professionalism OR professional behavio*) OR (MH "Health Personnel+"))

AND

(TI(competenc* OR cognizance OR cognisance OR knowledge OR skill* OR attitude* OR behavio*) OR AB(competenc* OR cognizance OR cognisance OR knowledge OR skill* OR attitude* OR behavio*) OR (MH "Attitude of Health Personnel+") OR (MH "Professional Knowledge+") OR (MH "Knowledge") OR behavior[mesh] OR (MH "Professional Competence") OR (MH "Clinical Competence+"))

AND

(TI(perception OR perceptions OR learning OR behavioral change* OR behavioural change* OR quality performance OR organizational performance OR organisational performanceOR patient outcome* OR professional development OR competentional development OR competence development OR awareness) OR AB(perception OR perceptions OR learning OR behavioral change* OR behavioural change* OR quality performance OR organizational performance OR organisational performanceOR patient outcome* OR professional development OR competentional development OR competence development OR awareness) OR (MH "Quality of Health Care+") OR (MH "Lifelong Learning") OR (MH "Skill Acquisition") OR (MH "Transfer (Psychology)") OR (MH "Learning") OR (MH "Reflection") OR (MH "Self-Awareness"))

**Cochrane**

| #1 | (“peer observation” OR “peer clinical observation” OR “direct observation” OR shadowing OR “peer feedback” OR “peer assessment”):ti,ab,kw (Word variations have been searched) | 1750 |
| --- | --- | --- |
| #2 | MeSH descriptor: [Formative Feedback] explode all trees | 82 |
| #3 | MeSH descriptor: [Peer Group] explode all trees | 1333 |
| #4 | MeSH descriptor: [Peer Review] explode all trees | 109 |
| #5 | MeSH descriptor: [Observation] explode all trees | 185 |
| #6 | #2 OR #3 OR #4 | 1511 |
| #7 | #5 AND #6 | 3 |
| #8 | #1 OR #7 | 1753 |
| #9 | (profession OR pro OR expert OR professionalism OR “professional behavior” OR “professional behaviour”):ti,ab,kw (Word variations have been searched) | 42859 |
| #10 | MeSH descriptor: [Health Personnel] explode all trees | 8386 |
| #11 | #9 OR #10 | 49602 |
| #12 | #8 AND #11 | 164 |
| #13 | (competenc* OR cognizance OR cognisance OR knowledge OR skill* OR attitude* OR behavio*):ti,ab,kw (Word variations have been searched) | 158674 |
| #14 | MeSH descriptor: [Health Knowledge, Attitudes, Practice] explode all trees | 5745 |
| #15 | MeSH descriptor: [Behavior] explode all trees | 85246 |
| #16 | MeSH descriptor: [Professional Competence] explode all trees | 3385 |
| #17 | #13 OR #14 OR #15 OR #16 | 204849 |
| #18 | #12 AND #17 | 102 |
