# Supplementary figures and images for: The application of the tracer method with peer observation and formative feedback for professional development in clinical practice: a scoping review
Source: Perspect Med Educ. 2021 Nov 11;11(1):15–21. doi: 10.1007/s40037-021-00693-6 (PMC8733089; doi:10.1007/s40037-021-00693-6)

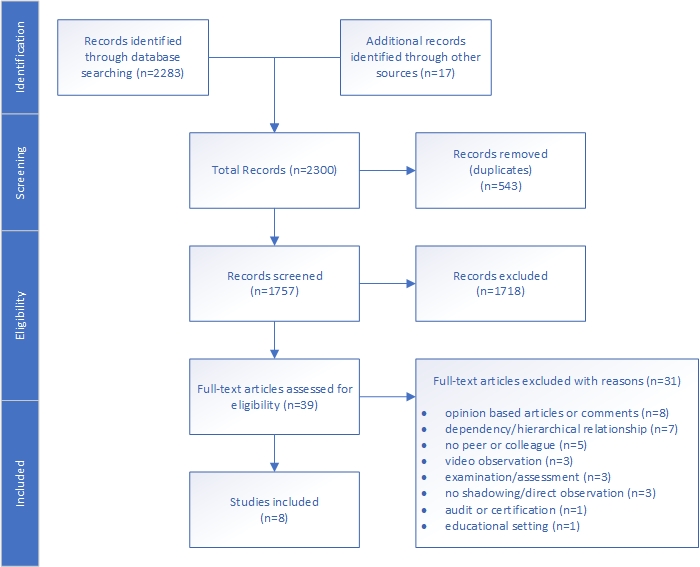


Figure 1: Flow Chart of the selection process

Supplement: Supplementary file 2 — Fig. 1 Flow Chart of the selection process [file 40037_2021_693_MOESM2_ESM.docx]
