# Supplementary material for: The application of the tracer method with peer observation and formative feedback for professional development in clinical practice: a scoping review
Source: Perspect Med Educ. 2021 Nov 11;11(1):15–21. doi: 10.1007/s40037-021-00693-6 (PMC8733089; doi:10.1007/s40037-021-00693-6)
Supplement: Supplementary file 3 — Table 1 Characteristics of the included studies [file 40037_2021_693_MOESM3_ESM.docx]

| **Characteristics** | **Reference** | | | | | | | |
| --- | --- | --- | --- | --- | --- | --- | --- | --- |
|  | Bhansali (2018) | Blumenthal (2019) | Borus (2018) | Bouma (2012) | Di Rocco (2020) | van Dulst (2016) | Lalleman (2017) | McDaniel (2019) |
| ***Study Location*** | | | | | | | | |
| Hospital | x |  | x | x | x | x | x | X |
| General Practice |  | x |  |  |  |  |  |  |
| ***Country*** | | | | | | | | |
| USA | x |  | x |  | x |  |  |  |
| Germany |  | x |  |  |  |  |  |  |
| Netherlands |  |  |  | x |  | x | x |  |
| Canada |  |  |  |  |  |  |  | X |
| ***Population*** | | | | | | | | |
| ***Participants (n)*** | **13** | **?** | **8** | **8** | **17** | **?** | **8** | **198** |
| Paediatric Hospitalists | x |  |  |  | x |  |  | X |
| Medical Doctors |  |  | x |  |  |  |  |  |
| General Practitioners |  | x |  |  |  |  |  |  |
| Nurse Managers |  |  |  | x |  |  | x |  |
| Nurse Practitioners |  |  | x |  |  |  |  |  |
| Healthcare Professionals |  |  |  |  |  | x |  |  |
| ***Type of Intervention*** | | | | | | | | |
| Peer Observation and Feedback (POF) | x | x | x |  | x |  |  | x |
| ***Purpose of Intervention*** | | | | | | | | |
| To describe experience with POF | x |  | x | x |  |  |  | x |
| To change professional attitude/behaviour |  | x |  | x | x |  |  |  |
| To investigate impact on learning/development |  |  |  |  |  | x | x |  |
| To assess efficacy |  |  | x |  |  |  |  |  |
| To characterize practice of POF |  |  |  |  |  |  |  | x |
| To identify preferences of POF |  |  |  |  |  |  |  | x |
| ***Methodology*** | | | | | | | | |
| Qualitative Design | x | x |  | x |  | x | x |  |
| Mixed-Methods |  |  | x |  | x |  |  |  |
| Quantitative Design |  |  |  |  |  |  |  | x |
| ***Used methodological Techniques*** | | | | | | | | |
| Interviews | x |  |  | x | x | x | x |  |
| Narrative |  | x |  |  |  |  |  |  |
| Survey/Questionnaire |  |  | x | x | x |  |  | x |
| Document study |  |  |  |  |  | x |  |  |
| Observation |  |  |  |  |  | x |  |  |
| ***Types of evidence (Kirkpatrick)*** | | | | | | | | |
| Reaction | x | x | x | x | x | x | x | x |
| Learning | x | x | x | x | x | x | x |  |
| Behaviour |  | x |  |  | x |  | x |  |
| Results |  |  |  |  | x |  |  |  |

*Table 1; Characteristics of the included studies*
